# The Role of a Confined Space on the Reactivity and Emission Properties of Copper(I) Clusters

**DOI:** 10.3389/fchem.2022.829538

**Published:** 2022-05-05

**Authors:** Eko Adi Prasetyanto, Youssef Atoini, Loic Donato, Chien-Wei Hsu, Luisa De Cola

**Affiliations:** ^1^ Institut de Science et D’Ingénierie Supramoléculaires (ISIS - UMR 7006), Université de Strasbourg, CNRS, Strasbourg, France; ^2^ Department of Pharmacy, School of Medicine and Health Sciences, Atma Jaya Catholic University of Indonesia, Jakarta, Indonesia; ^3^ Institute of Nanotechnology (INT), Karlsruhe Institut of Technology (KIT), Karlsruhe, Germany

**Keywords:** luminescence, confined space, copper clusters, zeolites, Cu(I), color tunability

## Abstract

Metal clusters have gained a lot of interest for their remarkable photoluminescence and catalytic properties. However, a major drawback of such materials is their poor stability in air and humidity conditions. Herein we describe a versatile method to synthesize luminescent Cu(I) clusters inside the pores of zeolites, using a sublimation technique with the help of high vacuum and high temperature. The porous materials play an essential role as a protecting media against the undesirable and easy oxidation of Cu(I). The obtained clusters show fascinating luminescence properties, and their reactivity can be triggered by insertion in the pores of organic monodentate ligands such as pyridine or triphenylphosphine. The coordinating ligands can lead to the formation of Cu(I) complexes with completely different emission properties. In the case of pyridine, the final compound was characterized and identified as a cubane-like structure. A thermochromism effect is also observed, featuring, for instance, a hypsochromic effect for a phosphine derivative at 77K. The stability of the encapsulated systems in zeolites is rather enthralling: they are stable and emissive even after several months in the air.

## Introduction

Currently, rare-earth-based phosphors are frequently used as emitters in lighting applications. (Bünzli and Piguet, 2005; Eliseeva and Bünzli, 2010). They are commercially available materials that are sufficiently photostable upon UV or blue excitation and meet all the industrial requirements regarding emission colors and efficiency. However, their very high cost and limited natural availability led researchers to find valuable alternatives. In this regard, various materials such as quantum dots based on zinc and cadmium selenides, (Raman et al., 1996; Frecker et al., 2016), have been developed with favorable spectral characteristics to replace rare-earth-based phosphors. Unfortunately, these are usually highly toxic and expensive, (Lewinski et al., 2008), and their use is not desirable. A valuable, cheaper, and a more environmentally friendly alternative is the use of metal complexes (Armaroli et al., 2007; Costa et al., 2012; Keller et al., 2018; Weber et al., 2018) and in this respect, silver has resulted in excellent luminophores with outstanding emission quantum yields when the clusters are protected by the environment. (Fenwick et al., 2016; Baekelant et al., 2019; Baekelant et al., 2020).

Amongst the use of cheaper and more abundant metals, copper is an interesting choice and many compounds have been investigated for their outstanding emission properties. (Armaroli et al., 2007; Costa et al., 2012; Keller et al., 2018; Weber et al., 2018). Indeed, M. Thompson *et al.* recently reported Cu(I) complexes reaching almost quantitative emission quantum yields. (Hamze et al., 2019; Shi et al., 2019). Nevertheless, their stability is still a critical issue and easy oxidation as well as distortion of their geometry in the excited state are the main drawbacks of such systems. (Itoh et al., 2002; Li et al., 2006; Brühwiler et al., 2009). A possible solution to decrease the non-radiative decay is to take advantage of a pronounced thermally activated delayed luminescence exhibited by some of these complexes. (Palmer and McMillin, 1987; Deaton et al., 2010; Linfoot et al., 2014; Baranov et al., 2020). This process consists in the back population of the lowest excited singlet state from the triplet luminescent level. This process could also solve the long-lived radiative emission decay for the T_1_→S_0_ transitions of Cu(I) compounds, typically in the range of hundreds of μs up to ms. (Armaroli et al., 2007; Costa et al., 2012; Keller et al., 2018; Weber et al., 2018). Usually, compounds with such a long excited-state lifetime would not be well suitable as emitters in devices or in solution since quenching processes such as *e.g.,* triplet-triplet annihilation or bimolecular deactivation can easily occur.

In addition, simple compounds such as Cu(I) clusters, (Kyle et al., 1991), containing halides as bridging ligands between the Cu(I) ions, are of great potential as emitters. Still, their formation and existence are restricted to particular conditions. From such versatile species, it is possible to generate a series of emissive compounds by simple substitution of the halide with coordinating ligands such as pyridines or phosphines. (Liu et al., 2012; Parmeggiani and Sacchetti, 2012; Benito et al., 2014).

Indeed, previous work has shown that three main products from reactions of CuI and pyridine, namely [CuI(py)]_2_ [CuI(py)_2_]_2_, and [CuI(py)]_4_ (py = pyridine), can be formed, showing blue, green, and orange emission, respectively, in the solid-state at room temperature. (Ford et al., 1999). Phosphine molecules have also been investigated as strong ligands in the synthesis of metal clusters due to the excellent coordination properties of phosphine that can stabilize metal clusters. (Perruchas et al., 2011). In general, a way to stabilize transition metal complexes (*i.e.,* preventing oxidation and distortion) can be performed by their encapsulation inside porous nanocontainers. For instance, the successful insertion of transition metal complexes inside mesoporous silica nanoparticles was already performed by De Cola *et al.*, Costa *et al.* and Hofkens *et al.*, using Ir(III) (de Barros e Silva Botelho et al., 2011; Ezquerro et al., 2019) Pt (II) (Atoini et al., 2018) and Cu(I)complexes (Donato et al., 2018) and Ag(I) clusters. (Fenwick et al., 2016).

This work presents an alternative strategy to stabilize the Cu(I) clusters by their encapsulation in inert silica-based porous materials. We demonstrate that a straightforward sublimation of copper iodide into the pores leads to the formation of luminescent clusters. In addition, the diffusion of a ligand inside the pores results in fast coordination to the Cu(I) ion and a change in the emission color are observed.

## Results and Discussion

**SCHEME 1:**
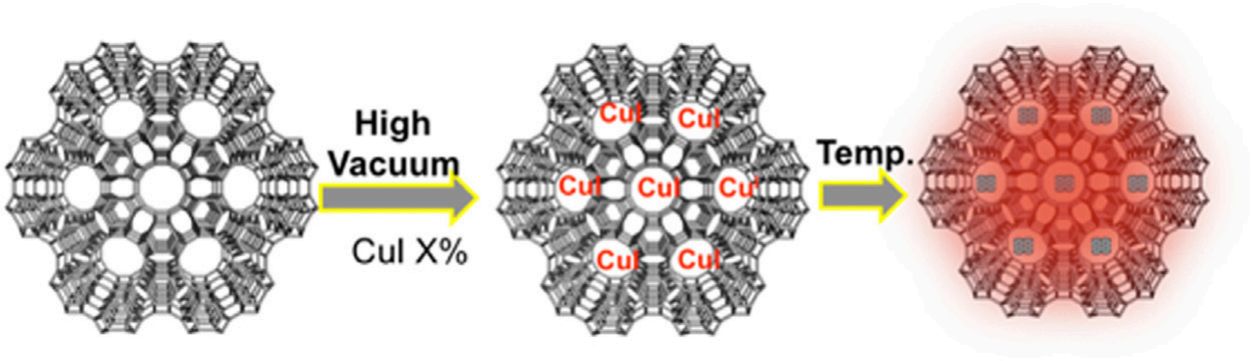
Schematic illustration of the synthesis of CuI cluster inside zeolite.

Porous silica-based materials such as zeolite L, zeolite Y, MCM-41, and SBA-15 were selected as convenient nanocontainers to be investigated as hosts for the formation of luminescent copper iodide clusters. The different materials differ in crystallinity, alumina content, charge, pore size, and order. For this work, the most relevant feature is the pore size that can influence the size and stability of the resulting clusters and their loading. Moreover, it should also play a role in the stability of the Cu(I) species since bigger pores might allow faster oxidation of the metal cation into Cu(II) due to faster oxygen diffusion inside the pores. Zeolites L (disk-shaped, 1 µm) were first filled by CuI salt by sublimation in a closed system (see experimental section) at high vacuum (10^–9^ bar) and 200 °C for 2 h (Scheme 1). Upon cooling, we observed a deep red luminescence coming from the zeolite L crystals.

This preliminary observation rules out the emission of the CuI salt since its weak emission is centered at 415 nm (Supplementary Figure S1). On the other hand, the copper clusters containing iodine as bridging ligand display a red emission similar to the one we recorded. It is important to note that the material displays the same emission *in vacuo* and after air exposure. As previously mentioned, the porous material plays a role as a stabilizing agent against the oxidation of Cu(I) into Cu(II). Moreover, the sublimation technique is not effective with complexes and clusters based on heavier transition metal ions, such as for instance platinum (II) or iridium (III), even with a longer heating time. Therefore, we can point out the quasi exclusivity of this approach for copper-based species.

**FIGURE 1:**
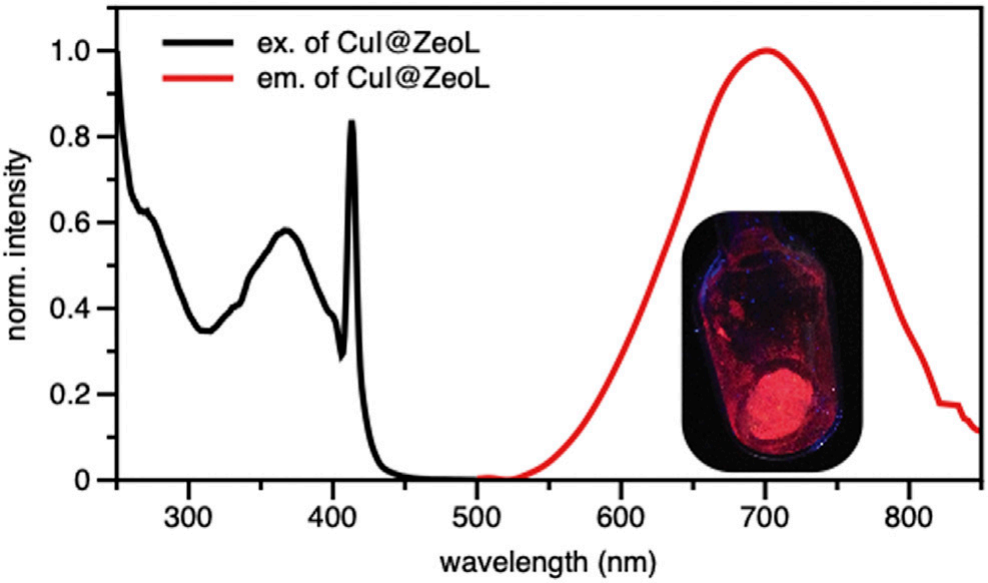
Excitation (λ_em_ = 700 nm) and Emission (λ_exc_ = 414 nm) spectra of CuI@ZeoL in solid-state condition; inset is the photograph of CuI@ZeoL inside the glass tube. The peak at 410 nm is an artifact.

In order to gain more insight into the photophysical properties of the species entrapped in the pores, excitation and emission spectra of CuI@ZeoL were recorded in the solid-state. The emission is characterized by a broad, non-structured band, with maximum of 710 nm (Figure 1) and an emission quantum yield of 14%.

The origin of the emission can only be assigned to a triplet “cluster-centered” (^3^CC) excited state, which has mixed iodide-to-metal charge transfer (^3^XMCT) and metal-centered transfer (^3^MC: d^10^ → d^9^S^1^Cu). The excitation spectrum recorded at λ_em_ = 710 nm shows a broad absorption at about 350 nm. The excited-state lifetime is a few microseconds (see Table 1), with complex multiple decay components pointing out the phosphorescence behavior of the material.

**TABLE 1:** Photophysiscal properties of the different hybrid materials.

Sample	λ_max_^Em^ (nm)	Φ_p_ ^a^ (%)/λ_ex_ (nm)	Lifetime 298K (μs)^b^		
			τ_1_ (μs)/%	τ_2_ (μs)/%	τ_3_ (μs)/%
CuI@ZeoL	712	14/360	5.2/6	1.01/27	0.17/67
CuI@ZeoY	711	8/360	4.13/10	1.1/45	0.24/45
CuI@MCM-41	711	7.5/360	6.25/35	0.65/65	−/−
CuI@SBA-15	712	5.7/360	5.76/27	0.97/73	−/−
CuIPy@ZeoY	575	34/360	11/90	2.37/10	−/−
CuIPy@ZeoY (77K)	435	n.a	22.7/100	−/−	−/−
CuIPPh3@ZeoY	695	12/412	8/70	1.87/26	0.34/4
CuIPPh3@ZeoY (77K)	493	n.a	2.5/72	0.29/28	−/−

a Absolute quantum yield was determined using an integrated sphere.

b Measured at λem = λmax (i.e. CuI@ZeoL, CuI@SBA-15: λem = 712 nm; CuI@ZeoY, CuI@MCM-41: λem = 711 nm; CuIPy@ZeoY: rt: λem = 575 nm, 77K: λem = 435 nm; CuIPPh3@ZeoY: rt: λem = 695 nm, 77K: λem = 493 nm).

**FIGURE 2:**
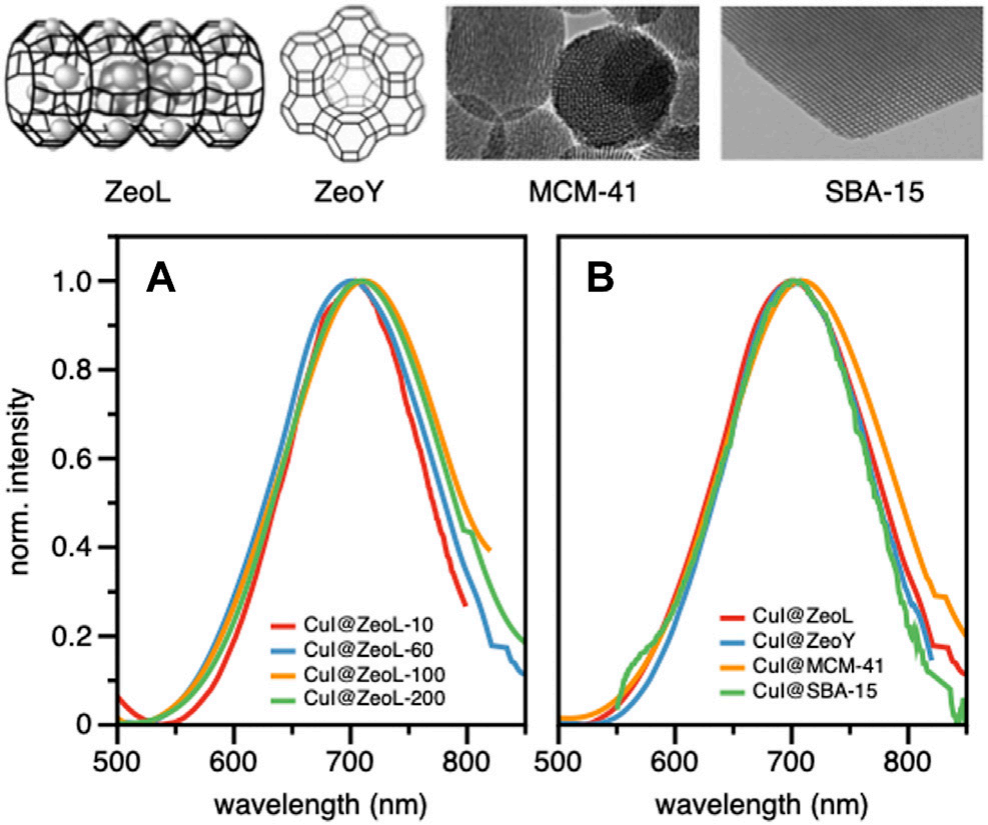
Structure and TEM image of the different porous materials used in this work (top); emission spectra of CuI@ZeoL with different ratios of CuI:ZeoL (CuI@ZeoL-A red line; CuI@ZeoL-B: blue line; CuI@ZeoL-C: orange line; CuI@ZeoL-D: green line) **(A)**; effect of host/pore size to the emission spectra **(B)** in solid-state condition. On top of the graphs is depicted the representation of the host used in Figure 2B λ_exc_ = 414 nm.

Confocal microscopy images (Supplementary Figure S2) clearly show that the emission originates from the zeolite crystals, emphasizing the successful encapsulation of CuI inside ZeoL, and the formation of a new emissive species. Moreover, different ratios in weight of CuI and ZeoL were studied (see supporting information) obtained by subliming different amounts of CuI in the same amount of zeolite (100 mg). In particular the ratios 10:100, CuI@ZeoL-A; 60:100 CuI@ZeoL-B; 100:100 CuI@ZeoL-C, after indicated only as CuI@ZeoL, and 200:100 CuI@ZeoL-D were prepared and the emission spectra of the obtained materials are depicted in Figure 2A. These data show that the emission energy is independent from the amount of CuI added to the zeolite.

Through the SEM images we can see that the morphology of the zeolite and silica material did not change before and after loading the copper source (Supplementary Figure S3), indicating that the loading of CuI did not destroy the porous structure of our materials. Meanwhile, we have obtained a red emission from the loaded CuI inside each host, which indicated that the copper source was successfully loaded inside the porous material. The photophysical properties of the resulting hybrid materials are discussed below.

To prove the confinement effect dictated by the porous containers, needed to obtain such a red emissive material, copper iodide powder was treated alone in the same manner (high vacuum and temperature), and after the treatment, we could not observe any red luminescence from this material. Moreover, the same procedure was performed with bulk, non-porous silica (Supplementary Figure S1). We clearly see the same emission as CuI alone, *i.e.,* an emission maximum at around 415 nm. This emphasizes the importance of the pores rather than the substrate to obtain emissive Cu(I) clusters. On the side, we performed nitrogen sorption experiments to show that the zeolites are indeed filled.

A comparison between the zeolites before and after sublimation of copper iodide reveals a decrease of the pore width and confirms a certain loading of the zeolites.

To understand the effect of the pore size, we have sublimed 100 mg of CuI inside materials featuring bigger pores, namely Zeolite Y (1.2 nm pore), MCM-41 (2 nm pore size), and SBA-15 (10 nm pore size), see Supplementary Materials section. The MCM-41, having 2 nm pore size and 100 nm in diameter, (Trewyn et al., 2007), and the SBA-15 mesoporous silica (SujandiPrasetyanto and Park, 2008) were synthesized following a typical sol-gel micelle-templated process. All those sublimation processes lead to the same deep red luminescence. (Table 1; Figure 2B). These results indicate the similarity of the cluster’s structure, without any dependence on the host used. However, it is essential to note that the cluster inside bigger pores is less stable toward degradation since it allows a more important penetration of oxygen. In the two extreme cases, *i.e.,* zeolite L and SBA 15, the red luminescence of the former does not decrease after several months, while it only takes a few days for the latter to lose its luminescence due to the oxidation of the Cu(I). The loading of the two materials: CuI@ZeoL and CuI@ZeoY (both of them with a ratio CuI:Zeo 100:100), was determined using the ICP-MS technique. The loading of the latter (CuI@ZeoY, 28%) is higher than that of the former (CuI@ZeoL, 13%), and this difference can be easily explained by the difference in the pore size, larger in the case of ZeoY. However, we could not detect evident changes in the emission maximum or excited state lifetimes suggesting that the luminescent species are the same (see Table1).

Knowing that Cu(II) based species are not luminescent, we could assume that the Cu(I) cluster was not oxidized since our material is strongly luminescent. However, the hypothesis of undesirable partial oxidation cannot be disproved only by the use of photophysical studies. In order to ensure there is no Cu(II) in our material, the oxidation state of Cu was then investigated by X-ray photoelectron spectroscopy (XPS) measurements (Supplementary Figure S5). As a control, pure CuI gives us the Cu2p_3/2_ peak at 932.9eV and I3d_5/2_ at 620.0eV. The spectra from Cu clusters show the Cu2p_3/2_ peak at 933.5eV and I3d_5/2_ at 620.9eV. The binding energy of copper stays in the Cu(I) region compared to the other copper halogen compounds. But more importantly, the spectrum does not feature any signal around 960eV, typical for Cu(II) (satellite signal of Cu(II)).

Both the Cu and I peaks shifted to a little bit higher binding energy in the clusters, which is due to the interactions with the oxygen atoms on the zeolite channels. All the signals present only one contribution in agreement with only one chemical environment, indicating that there is no oxidation of Cu(I).

**FIGURE 3:**
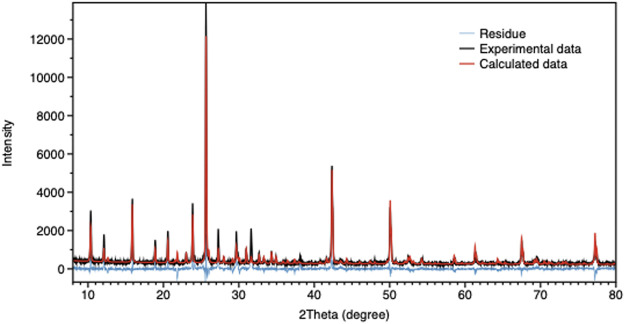
TOPAS calculation result for CuI@ZeoL. The black curve shows the measured XRD data, the red curve shows the calculated Rietveld fitting, and the blue curve shows the difference between the former and the latter.

To assess the structure of the CuI cluster, the species formed inside the zeolite were analyzed by powder X-ray diffraction (PXRD), and the results were determined by Total Pattern Analysis Solutions (TOPAS) software using advanced Rietveld refinement. (Coelho, 2003). Quantitative phase analysis QPA in the TOPAS software is based on the method first described by Hill and Howard. (Hill and Howard, 1987). This method is based on the assumption that 1) all phases in the specimen are identified, 2) all phases are crystalline, and 3) the crystal structures of all phases are known. By comparing the experimental data, calculated data, and its residual (Figure 3), we found that the Cu(I) clusters inside the zeolite have cubic type crystal structure with F-43m space group and a = b = c = 6.056 Å, featuring a Rietveld weigh profile (Rwp) value of 7.8. By performing calculations, we obtained the crystal symmetry, lattice parameter, and we could also determine the atomic composition of the cluster. Finally, we were able to reconstruct the crystal structure of the CuI cluster, as shown in Supplementary Figure S6. The position of the copper cluster inside the zeolite Y was then reconstructed and shown in Supplementary Figure S7.

Two different organic ligands, *i.e.,* pyridine and triphenyl-phosphine, were inserted in CuI@porous materials. As explained here above, porous hosts have small pores. In other words, zeolites are better candidates for stability reasons.

In this work, zeolites L present too small channels to host the organic ligands. For these reasons, the material chosen for the following of this work is zeolite Y, most likely the best candidate for such a purpose. (Yu et al., 2014; Łukarska et al., 2017). After the addition of the organic ligands into CuI@ZeoY, the characterization of the resulting materials was performed. In order to determine the structure of the resulting CuI cluster formed in zeolites, an interesting approach was to use PXRD analysis by comparing.

One of the materials we obtained with a simulation of the addition of zeolite Y and the already.

**SCHEME 2:**
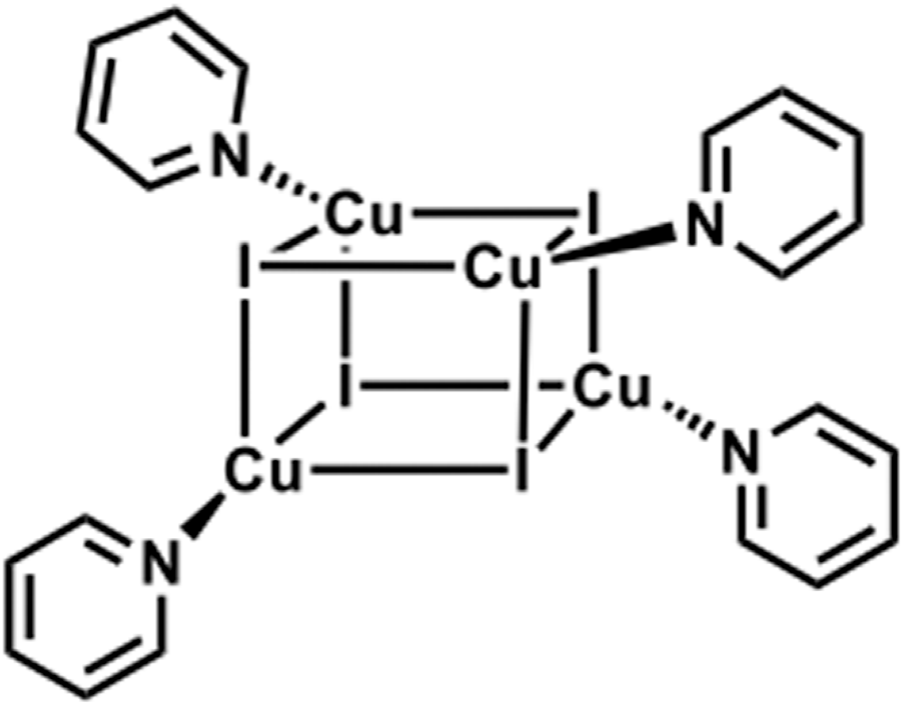
Schematic representation of CuI cluster functionalized with pyridine ligands featuring a cubane-like structure. (Ford et al., 1999).

Reported (C_5_H_5_CuIN)_4_ cubane cluster (Supplementary Figure S8). (Raston and White, 1976). PXRD studies showed a high degree of crystallinity of the materials, and again by using TOPAS software, (Coelho, 2003), we were able to identify our guest cluster as a cubane-like structure (Scheme 2). It is important to mention the matching between the experimental and the measured patterns, and the Rwp found is 6.5, which is a very good value for such studies.

To confirm this result, the compound was isolated from the zeolites by breaking the latter by means of a sonication process. The resulting species was characterized by ^1^H NMR, DOSY NMR, and HR ESI-MS. NMR spectra of the CuIPy cluster were compared with that of pyridine. While ^1^H NMR of both molecules, *i.e.*, CuIPy cluster and pyridine, have the same protons, they display similar spectra, featuring three signals in the aromatic region; the main difference relies on a downfield shift of the signals referring to CuIPy cluster, by around 0.5 ppm (Supplementary Figure S9).

DOSY NMR spectra (Supplementary Figure S10, Supplementary Table S1) show a diffusion coefficient of 1.46 nm s^−1^ and 2.93 nm s^−1^ for CuIPy cluster and pyridine, respectively. Hence, the molecular volumes determined for pyridine and the CuIPy cluster are respectively 21 Å^3^ and 169 Å^3^.

HR ESI-MS spectrum (Supplementary Figure S11) shows an m/z value of 1,075.51, matching perfectly the theoretical predictions of Cu_4_I_4_(C_6_H_5_N)_4_. Thus, the isolated molecule was confirmed to be (CuIPy)_4_.

Copper-iodine clusters were intensively studied by Peter C. Ford. In this regard, a cubic copper-iodine bearing a pyridine on each copper atom was characterized. (Liu et al., 2012). Inline of Ford's studies, our sublimation process, followed by pyridine addition, aims to obtain the same cluster inside the zeolites Y pores.

**FIGURE 4:**
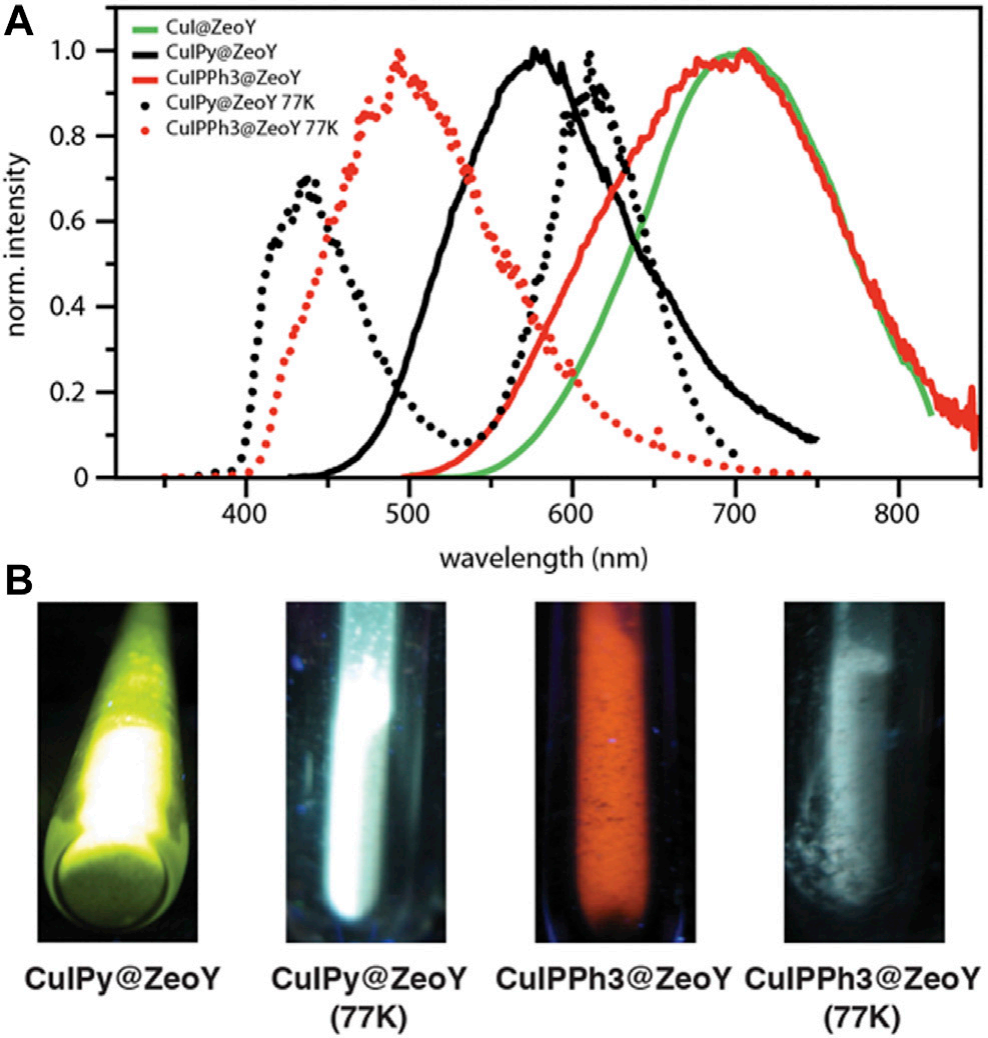
Emission spectra of CuI@ZeoY in solid-state condition; λ_exc_ = 414 nm.

It has been noticed that both clusters containing organic ligands, *i.e.,* CuIPy@ZeoY and CuIPPh_3_@ZeoY, feature a thermochromic behavior. In fact, in thermochromic clusters, the luminescent thermochromism is caused by a relative intensity change of two distinct emission bands. (Kitagawa et al., 2010; Perruchas et al., 2010; Fenwick et al., 2016; Tsuge et al., 2016; Baekelant et al., 2019; Baekelant et al., 2020). Hence, the emission of the materials was also measured at 77K (Figure 4), and a correlation could be found between the temperature and the emission profiles. Indeed, the high energy emission band at around 400–450 nm is dominant at low temperature, assigned to a halogen-to-ligand charge transfer (XLCT) transition state.

The low energy emission band at around 575 nm for CuIPy@ZeoY and 695 nm for CuIPPh_3_@ZeoY is dominant at room temperature, which is referred to a cluster-centered excited state (^3^CC) and can be affected by outer environments such as solid/solution state, temperature, or pressure. Regarding CuIPy@ZeoL, the photophysical data match perfectly with the cluster previosuly described eliminate in Peter Ford studies, this allows us to confirm the cubane like structure, referring to the following chemical formula: (Cu_4_I_4_Py_4_). (Kyle et al., 1991; Ford et al., 1999).

When the tube was taken out from liquid nitrogen and exposed to the irradiation of a UV lamp (365 nm), the interesting reversible thermochromic luminescence from yellow (298 K) to blue (77 K) can be easily distinguished by the naked eye or recorded by a digital camera. Solid-state luminescence performed from 298 to 77 K is in accordance with the thermochromic luminescence and the variation of luminescent intensity. At room temperature, the maximum of the single emission is found at 575 nm with an excited state lifetime featuring a bi-exponential decay, 11 and 2.37 μs, and an emission quantum yield of 34% (Figure 4; Table 1). The long excited-state lifetime, in the microsecond range, indicates the phosphorescence behavior, which is not surprising coming from such clusters. With the decrease of temperature from room temperature to 77 K, the emission band is progressively blue-shifted from 575 to 435 nm, which is accompanied by a widening of the bandwidth and a decrease in emission intensity. This dramatic hypsochromic shift of about 140 nm makes the detection of the color change by the naked eye easier. Without the presence of an organic ligand, the origin of the emission can only be assigned to a triplet “cluster-centered” (^3^CC) excited state, which has mixed iodide-to-metal charge transfer (^3^XMCT) and metal-centered transfer (^3^MC: d^10^ → d^9^S^1^Cu). However, in the case of Cu_4_I_4_L_4,_ other transitions such as organic-ligand-related excited states, *i.e.,* halide to ligand charge transfers (XLCT), must be considered.

Moreover, it has been shown that the thermochromic luminescence is due to a significant change in Cu⋯Cu distances that decreases together with the temperature. (Ford et al., 1999). Indeed, at lower temperatures, Cu⋯Cu distances become shorter, and the bonding character becomes more and more obvious. The transition of the LT phase to the HT phase resulted in some shortening of Cu⋯Cu distances. The origin of thermochromic luminescence in CuI cluster is quite different from cubane Cu_4_I_4_L_4_ cluster-based compounds, where relative intensities of ^3^CC and XLCT emissions with the temperature are responsible for the thermochromic luminescence. (Tard et al., 2008).

## Conclusions

In conclusion, we have synthesized luminescent copper iodide inside porous nanomaterials, with a relatively high photoluminescence quantum yield. The nanomaterial allow high stability due to its protection from oxygen, preventing the oxidation of Cu(I) into Cu(II). A certain correlation was also found between the pore size and the stability of the Cu(I) cluster. By adding an organic monodentate ligand after sublimation of CuI, *i.e.,* pyridine or triphenylphosphine, we are able to tune the luminescence energy, the resulting luminescence featuring an important bathochromic shift. The materials also showed a thermochromic behavior, which led to a significant hypsochromic emission shift of 140 nm. Thanks to several characterizations, among them ESI-HRMS, DOSY NMR, and PXRD experiment followed by Rietveld refinement and simulation, we were able to identify Cu(I) cluster and CuIPy cluster, the latter having a cubane like structure. This finding may lead to the use of copper, much cheaper than its transition metals counterparts, as alternative materials in applications such as lighting devices.

## Data Availability

The original contributions presented in the study are included in the article/Supplementary Material, further inquiries can be directed to the corresponding authors.
