# Supplementary material for: The Role of a Confined Space on the Reactivity and Emission Properties of Copper(I) Clusters
Source: Front Chem. 2022 May 5;10:829538. doi: 10.3389/fchem.2022.829538 (PMC9117724; doi:10.3389/fchem.2022.829538)
Supplement: Supplementary file 1 [file DataSheet1.pdf]

Supplementary Materials

The role of a confined space on the reactivity and emission properties of copper(I) clusters

Eko Adi Prasetyanto,* [†,§,1] Youssef Atoini,[†,1] Loic Donato,[†] Chien-Wei Hsu,[†] and Luisa De Cola*[†,‡,$]

[†] Institut de Science et d'Ingénierie Supramoléculaires (ISIS - UMR 7006), Université de Strasbourg – CNRS. 8 Rue Gaspard Monge, F67000 Strasbourg, France.

[§] Dept. of Pharmacy, School of Medicine and Health Sciences, Atma Jaya Catholic University of Indonesia. JL. Pluit Raya no 2, Jakarta, Indonesia.

[‡] Institute of Nanotechnology (INT), Karlsruhe Institut of Technology (KIT), Hermann-von-Helmholtz-Platz 1, 76344 Eggenstein-Leopoldshaffen, Germany.

[$]Current address: Department of Pharmaceutical Sciences, University of Milan, Milan, 20133, Italy

[1] These authors have contributed equally to the article.

Email: luisa.decola@unimi.it, prasetyanto@atmajaya.ac.id

Table of content:

1. Instrumentations and Materials ……………………………………………... Page 1

1.1. Instrumentation ……………… ………………………………………… Page 1

1.2. Materials Preparation……..……………………………………………… Page 2

2. Figures……………………………………………………………………….. Page 4

1. Materials and Instrumentations

1.1. Instrumentation

All chemicals were commercially available and used without further purification.

Steady-state emission spectra were recorded on a Horiba Jobin−Yvon IBH FL-322 Fluorolog 3 spectrometer equipped with a 450 W xenon arc lamp, double-grating excitation, and emission

monochromators (2.1 nm mm^{-1} of dispersion; 1200 grooves mm^{-1}) and a TBX-04 single photon-counting detector. Emission and excitation spectra were corrected for source intensity (lamp and grating) and emission spectral response (detector and grating) by standard correction curves. Time-resolved measurements were performed using the time-correlated single-photon-counting (TCSPC) PicoHarp300 or the Multi Channel Scaling (MCS) electronics NanoHarp 250 of the PicoQuant FluoroTime 300 (PicoQuant GmbH, Germany), equipped with a PDL 820 laser pulse driver. A pulsed laser diode LDH-P-C-405 (λ_{exc}= 405 nm) was used to excite the sample and mounted directly on the sample chamber at 90°. The photons were collected by a PMA-C-192 photomultiplier(PMT) single-photon-counting detector. The data were acquired by using the commercially available software EasyTau (PicoQuant GmbH, Germany), while data analysis was performed using the commercially available software FluoFit (PicoQuant GmbH, Germany).

PLQY measurements were performed by using an absolute photoluminescence quantum yield spectrometer Quantaurus C11347 (Hamamatsu, Japan) exciting the sample at λ_{exc} = 350 and 400 nm. Confocal microscopy analyses were performed by using Zeiss LSM 710 confocal microscope system with 63X magnification, numerical aperture 1.3 of Zeiss LCI Plan-NEOFLUAR water immersion objective lens (Zeiss GmbH). The samples were excited by laser at 405 nm. All image processing was performed by ZEN 2011 software.

XPS measurements were done by a Thermo Scientific K-Alpha X-ray Photoelectron Spectrometer using a monochromatic AlKα radiation (hν= 1486.6 eV; λ= 8.340113Å). Element scans were performed with a 50 eV analyzer pass energy and a 0.1eV energy step size obtain the chemical state information. All the obtained binding energies were referenced from carbon 1s peak, coming from the residual CO_2, at 284.80eV.

^{1}H and DOSY NMR spectra were recorded on a Bruker Avance III 600MHz, using as solvent deuterated dichloromethane (CD_2Cl_2). The residual CH_2Cl_2 present in the solvent was used as internal standard (δ = 5.35 ppm).

Liquid-chromatography coupled with high-resolution mass spectrometry (HPLC-HRMS) was performed in positive mode using a ThermoFisher Ultimate3000 with Scientific Vanquish Flex UHPLC and a ThermoFisher Orbitrap (Exactive Plus with Extend Mass Range: Source HESI II The detector is a Vanquish PDA Detector (VF-XX, detection ≤ 5 ppm). Direct injection high-resolution mass spectrometry was used using as eluant HPLC grade dichloromethane 1 : 9 methanol.

Nitrogen Adsorption-Desorption analyses of the samples were performed using a Micromeritics porosimeter (model ASAP-2020). The samples were degassed at 150 °C for 3h and N2 adsorption/ desorption measurement was done at -196 °C. The surface areas and pore volume were calculated by BET method and the pore size distributions were calculated by DFT methods.

The loading ratio was determined using ICP-AES with a Varian 720 ES instrument at 324.754 nm for Cu. Quantification was performed by a calibration curve established with standards (0, 0.025, 0.1, 0.5, 2, 10 mg/L) prepared from certified standards (1000 mg/L; CPI International) after dilution of the samples.

1.2 Materials Preparation

Synthesis of MCM-41

In a 250ml round bottom flask Hexadecyl-trimethyl-ammonium bromide (CTAB; 0.5 g) was dissolved in 240 mL of Sodium hydroxide solution (0.00002 M) at 80 °C. Then, 2.5 mL of 0.88 M (1.833 g per 10 mL) ethanolic Tetraethyl orthosilicate (TEOS) and 3-aminopropyltriethoxysilane (APTES; 50 μl) was added to the solution under vigorous stirring. After 1 h, the mixture solution was aged for at least 12 h. Few drops of Hydrochloridric acid solution (6M) were added to the as-synthesized colloid with stirring at room temperature for 5 h, to remove surfactant. The surfactant extraction step was repeated several times to ensure removal of CTAB. The extracted MSNs were washed with ethanol twice and resuspended in absolute ethanol. The material was obtained as a white powder. (Final amount =185mg)

Synthesis of SBA-15

1.5 g [Poly(ethylene glycol)-block-poly(propylene glycol)-block-poly(ethylene glycol)] (Pluronic® P123) was dissolved into 42 mL water, then 4.36 g $Na_2SiO_3 \cdot 9H_2O$ and 0.292 mL APTES was added. The solution was then heated up to 40˚C with vigorous stirring. After the temperature was stable, 10.92 mL of 37% HCl was added and the stirring was kept at 40˚C for 1 h. The solution was transferred into a sealed glass bottle and kept at 100˚C for 24 h. The final product was centrifuged and washed by water 3 times, then dried in vacuum, and calcined at 550˚C for 6h to remove the remaining surfactant. The material was obtained as a white powder. (Final amount =550mg)

Synthesis of CuI clusters inside porous materials

The porous materials and the zeolites were always 100 mg, and placed inside a glass ampoule in the presence of the follow amount of CuI. For the Zeolite Y (1.2 nm pore), MCM-41 (2 nm pore size), and SBA-15 (10 nm pore size) 100 mg of CuI was sublimed. For the zeolite L different amount of CuI was sublimed and in particular the following amount of CuI was sublimed for the different samples:

A. 10 mg (0.052mmol)
B. 60 mg (0.31 mmol)
C. 100 mg (0.52 mmol)
D. 200 mg (1.04 mmol)

A vacuum was applied to the ampoule (10^{-9} bar), and the latter was sealed before being placed in a rotating oven. After heat treatment at 200°C for 2h together with a rotation of the tube, the sealed ampoule was opened, and, for each sample, a white, red emissive powder was obtained. Final amount obtained after synthesis: CuI@ZeoL-A: 74 mg; CuI@ZeoL-B: 98 mg; CuI@ZeoL-C: 122 mg; CuI@ZeoL-D: 168 mg

Loading of CuI inside the zeolites

The loading ratio was determined using ICP-AES with a Varian 720 ES instrument at 324.754 nm for Cu.

Num Lab	Si	Al	Mg	Ca	Fe	Mn	Ti	Na	K	P	Sr	Ba	V	Ni	Co	Cr	Zn	Cu	Li
Reference	*0.1*	*0.01*	*0.001*	*0.001*	*0.005*	*0.005*	*0.0002*	*0.09*	*0.2*	*0.003*	*0.001*	*0.0007*	*0.004*	*0.005*	*0.002*	*0.002*	*0.0006*	*0.0002*	*0.01*
CuI-ZeoY Material	15.3	11.2	0.064	0.142	0.050	-	0.0332	10.5	0.4	0.006	0.002	0.0009	-	-	-	0.003	0.0061	3.58	-
CuI-ZeoL Material	27.9	17.3	0.044	0.143	0.032	-	0.0131	18.0	-	-	-	-	-	-	-	-	0.0166	2.32	1.16

Zeolite Y crystal: (Na2, Ca, Mg)$_{3.5}$[$Al_7Si_{17}O_{48}$].32(H_2O)→ Mw=2393 g/mol; 7Al and 17Si

Zeolite L crystal: $K_6Na_3(H_2O)_{21}Al_9Si_{27}O_{72}$ → Mw =2835 g/mol; 9Al and 27Si

CuI: Mw=190 g/mol

- For CuI@ Zeolite Y Material:

- m Si= (15.3*2393)/17 = 2153 mg; m Cu=3.58*190= 680.2 mg → mass ratio= m Cu/ m Si = 680.2/2135 = 0.32 = 32%

- m Al= (11.2*2393)/7 = 3828 mg; m Cu=680.2 mg → mass ratio = m Cu/ m Al = 680.2/3828 = 0.18 = 18%

-mass ratio average of Si+ Al:

(0.32*17)+(0.18*7)/24=28%

Loading CuI@Zeolite Y= 28%

- For CuI@ Zeolite L Material:

-m Si= (27.9*2835)/27 = 2929mg ; m Cu= 2.32*190=440 mg → mass ratio = m Cu /m Si = 440/2929 = 0.15

-m Al= (17.3*2835)/9 = 5449 mg; m Cu= 2.32*190=440 mg →mass ratio= m Al/m Si= 440 mass ratio = m Cu /m Al = 440/5449= 0.08

-mass ratio-average of Si+ Al:

(0.15*27)+(0.08*9)/36 =0.132 = 13%

Loading CuI@Zeolite L= 13%

***Synthesis of* CuIPy clusters inside porous materials**

To a suspension of CuI inside porous materials (100 mg) inside ethanol (25 mL), was added pyridine (85 μL, 1.04 mmol) and the reaction mixture was stirred overnight at room temperature. The mixture was then centrifuged and washed 3 times with cold ethanol in order to yield the final material, CuIPy@porous material as an off-white and yellow emissive powder.

***Synthesis of* of CuIPPh$_3$ clusters inside porous materials**

To a suspension of CuI inside porous materials (100 mg) inside ethanol (25 mL), was added pyridine (273 mg, 1.04 mmol) and the reaction mixture was stirred overnight at room temperature. The mixture was then centrifuged and washed 3 times with cold ethanol in order to yield the final material, CuIPy@porous material as an off-white and yellow emissive powder.

2. Figures

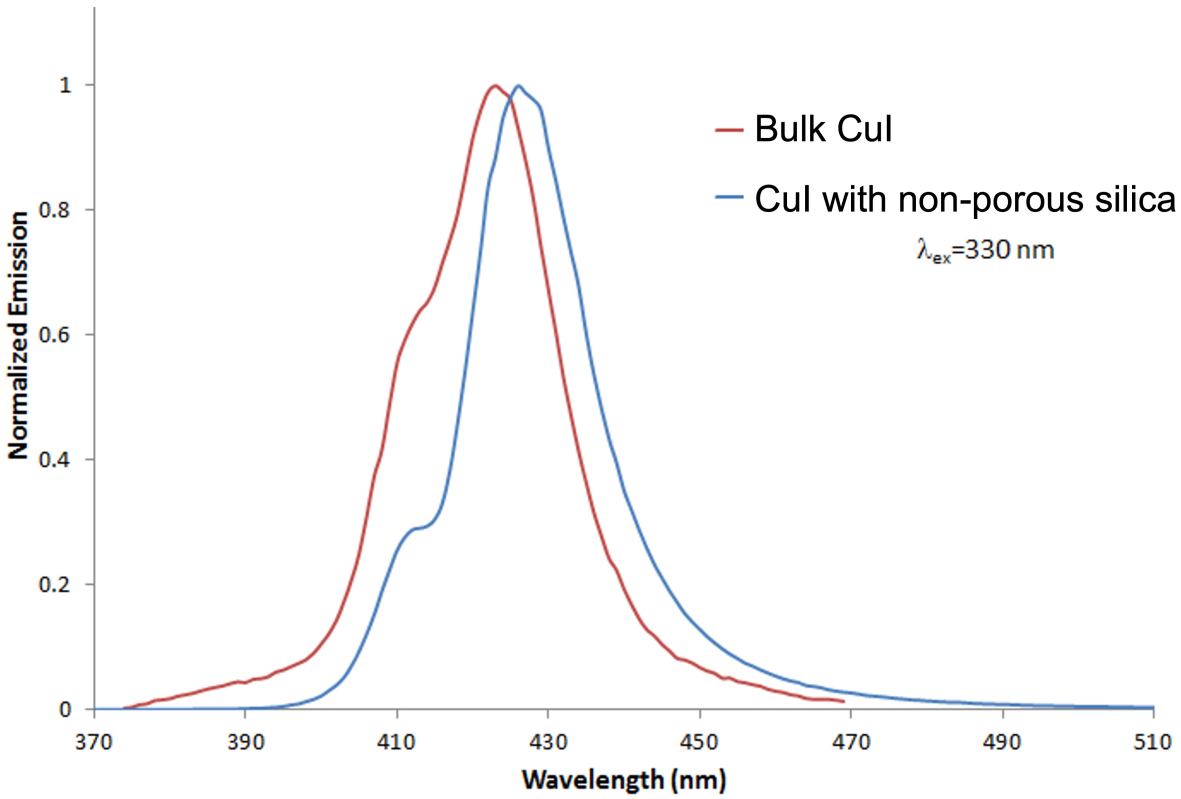

Figure S1. Comparison between emission of bulk copper iodide and copper iodide sublimed in presence of non-porous silica.

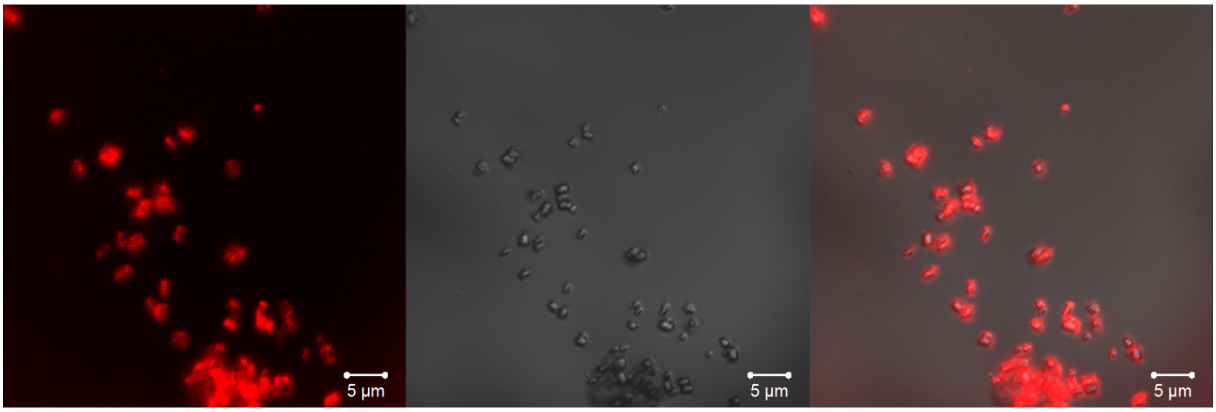

Figure S2. confocal microscopy image of CuI cluster inside Zeolite L.

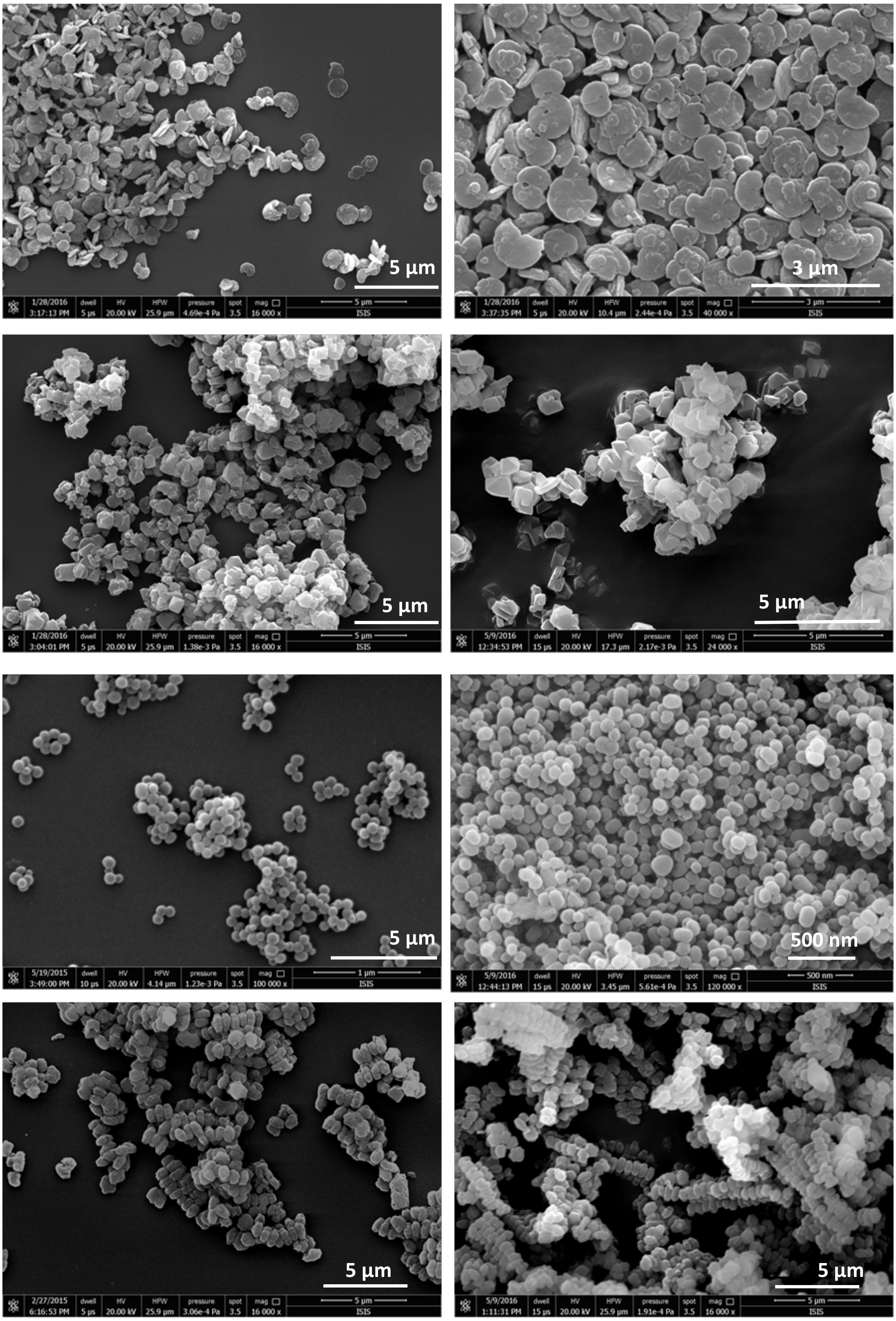

Figure S3. SEM images of zeolite LTL (Zeolite L), zeolite FAU (Zeolite Y), CTAB-silica (MCM-41), P123-silica(SBA-15) before (left) and after (right) the loading of CuI.

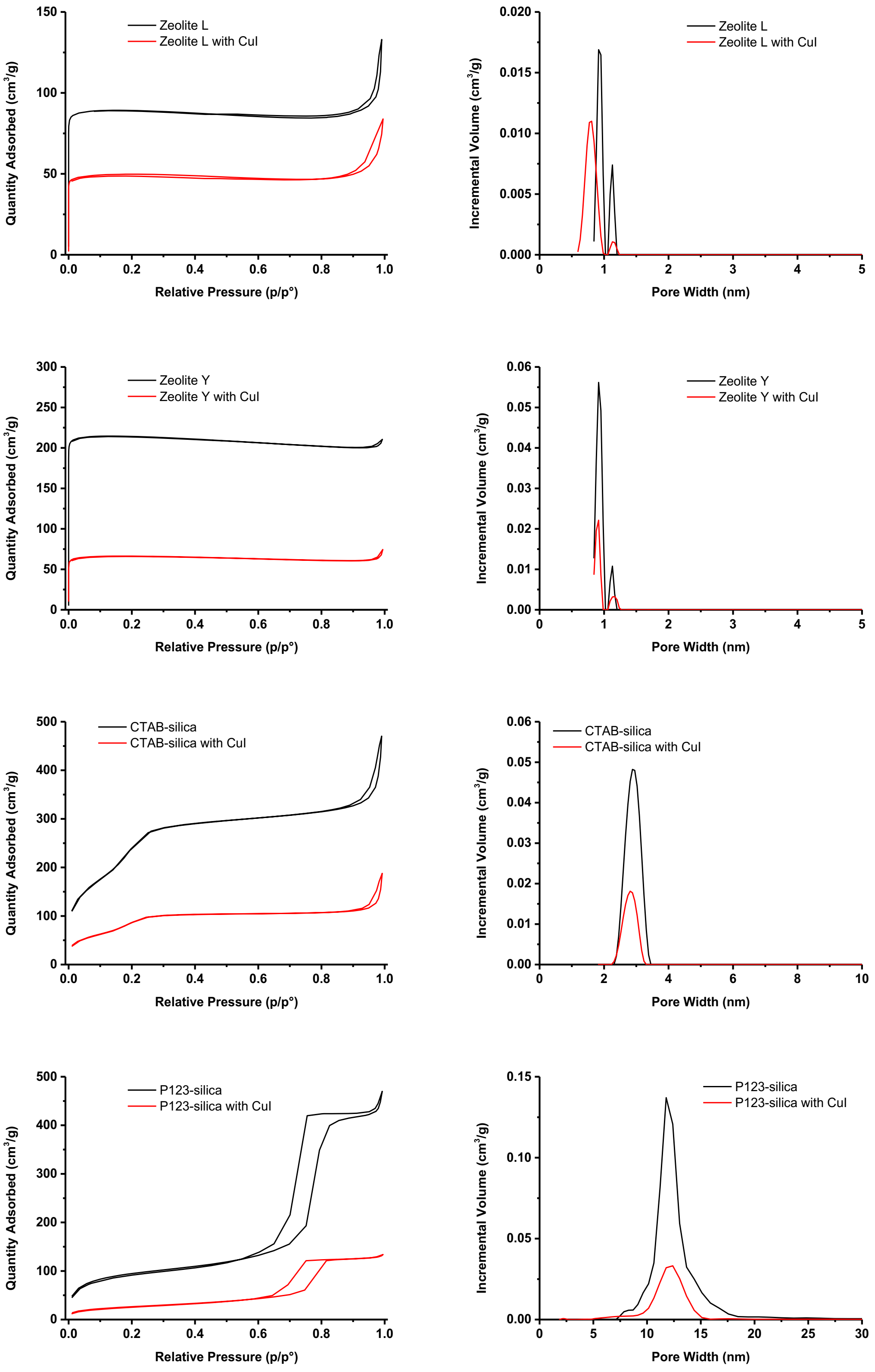

Figure S4. N_2 adsorption isothermal and pore distributions of different porous materials before and after sublimation of CuI into the pore.

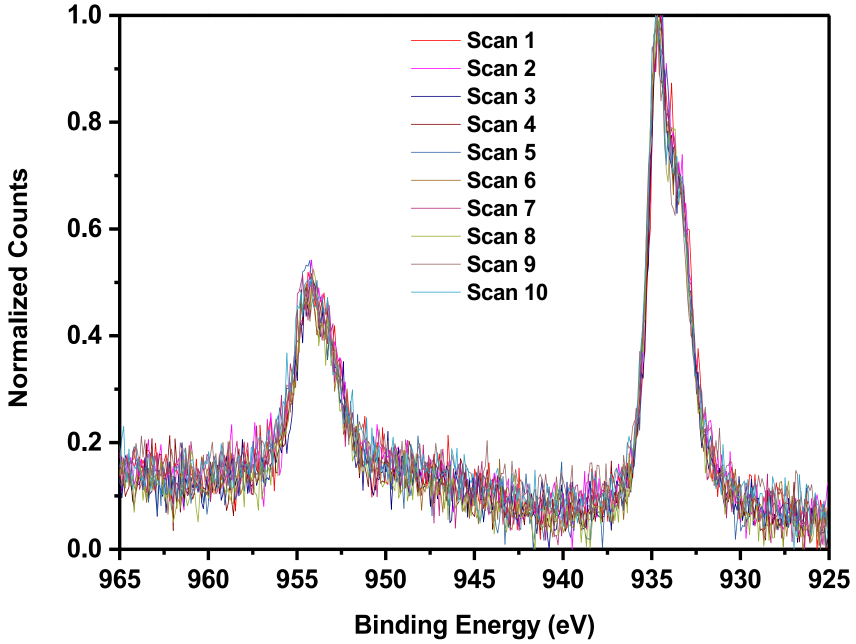

Figure S5. XPS spectra of the CuI loaded zeolite LTL sample.

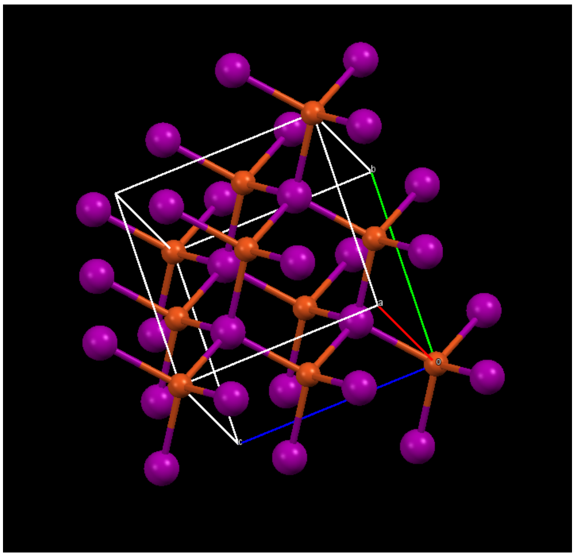

Figure S6. Simulated structure of CuI cluster.

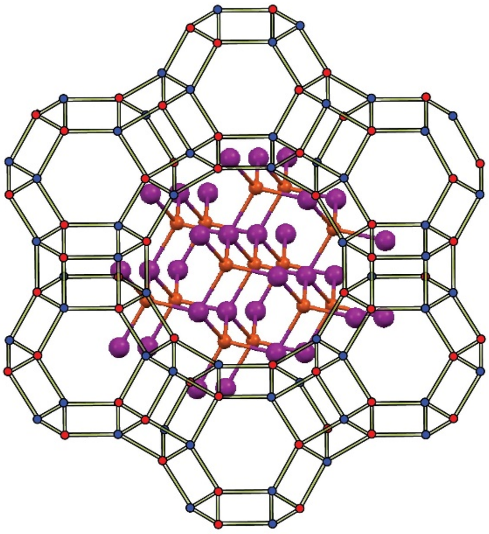

Figure S7. Illustration of simulated CuI cluster, with its position in the zeolite Y.

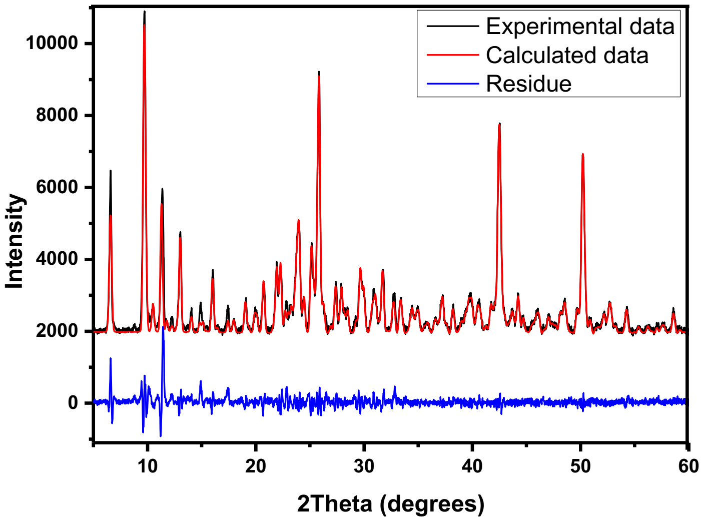

Figure S8. Powder XRD pattern of CuiPy@Zeo Y : Experimental data (black line), simulation of the addition between zeolite Y and $(C_5H_5CuIN)_4$ cubane cluster (red line) difference between the experimental and calculated patterns (blue line).

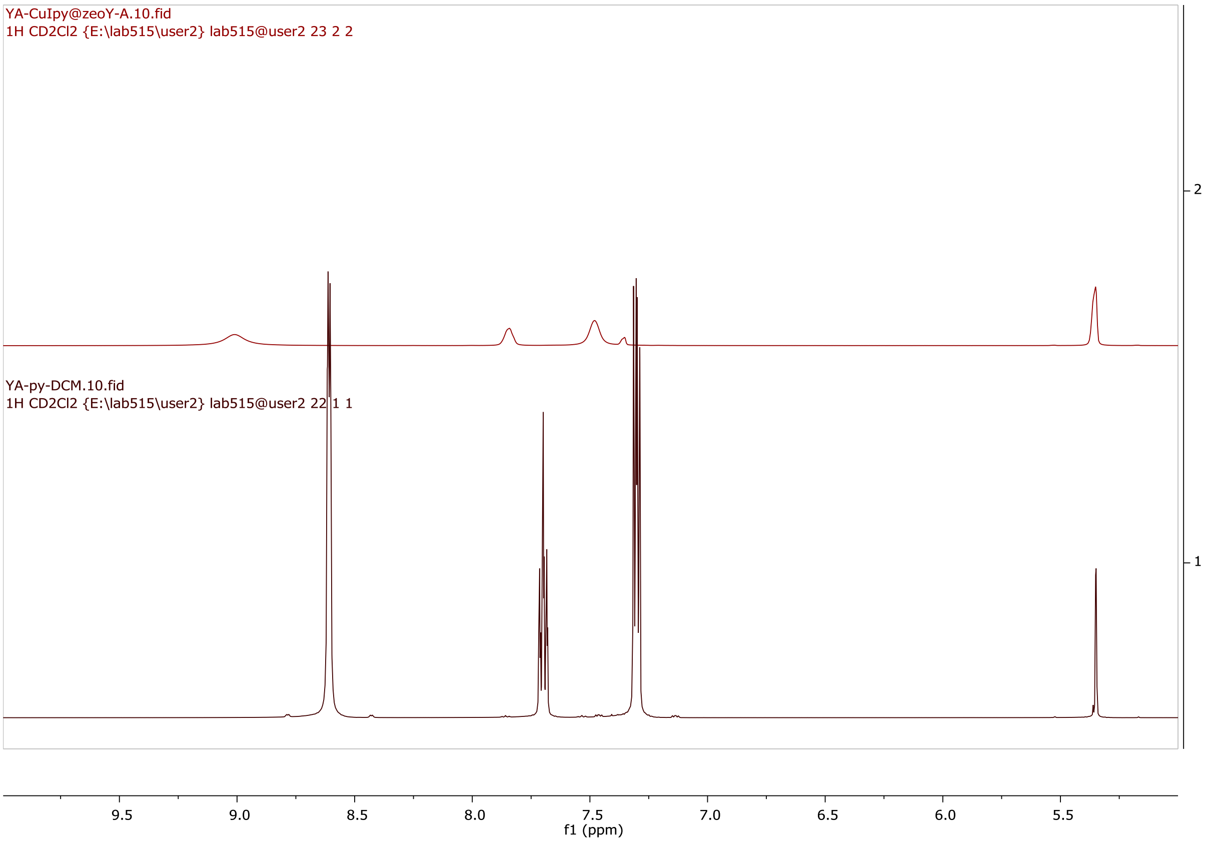

Figure S9. ^{1}H NMR spectrum of pyridine (bottom) and the isolated $(CuIPy)_4$ (top) at 298K in D_2 dichloromethane.

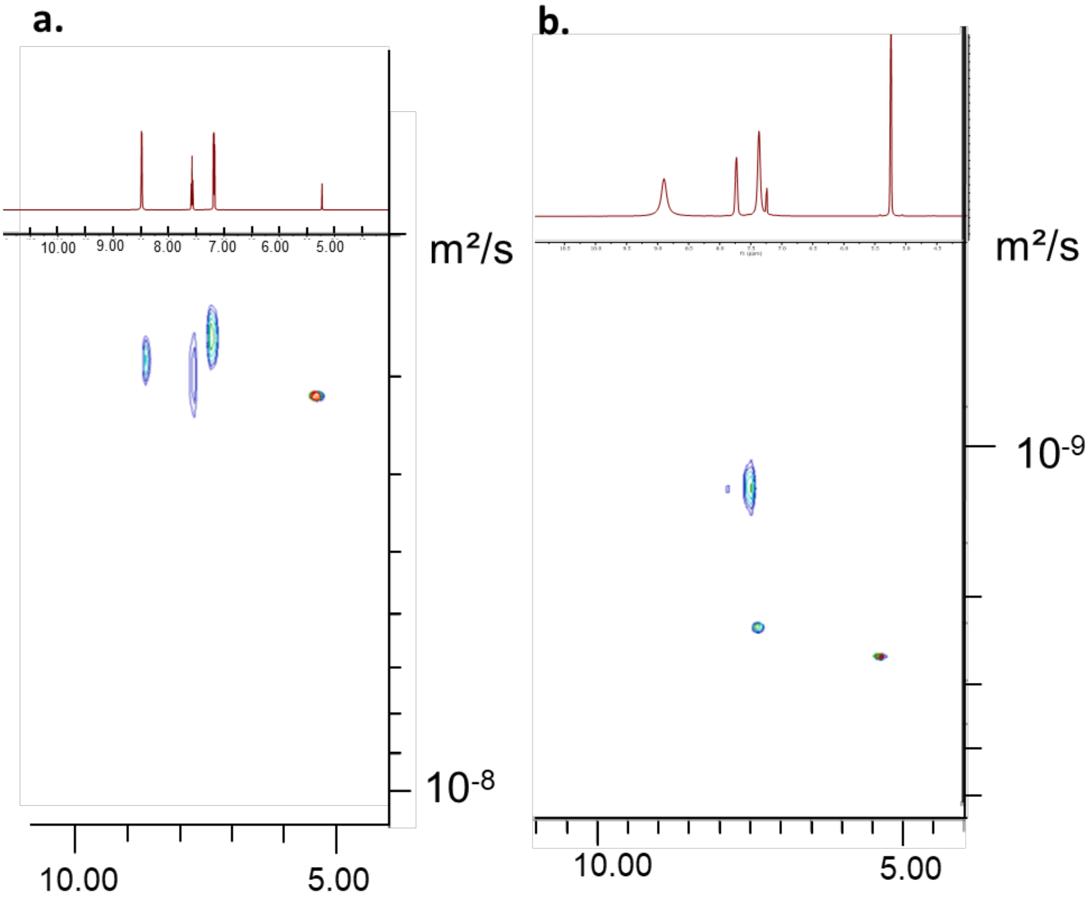

Figure S10. DOSY NMR spectrum of pyridine (a.) and the isolated $(CuIPy)_4$ (b.) at 298K in D_2 dichloromethane.

Table S1: DOSY NMR data of pyridine and $(CuIPy)_4$ at 298K (solvent: D_2 dichloromethane).

	Diffusion coefficient D ($m^2 \cdot s^{-1}$)	Viscosity μ (Pa·s)	Hydrodynamic radius R_h	Molecule's volume V (Å^3)
Pyridine	$2.93 \cdot 10^{-9}$	$4.353 \cdot 10^{-4}$	$1.71 \cdot 10^{-10}$	21
$(CuIPy)_4$	$1.46 \cdot 10^{-9}$	$4.353 \cdot 10^{-4}$	$3.43 \cdot 10^{-10}$	169

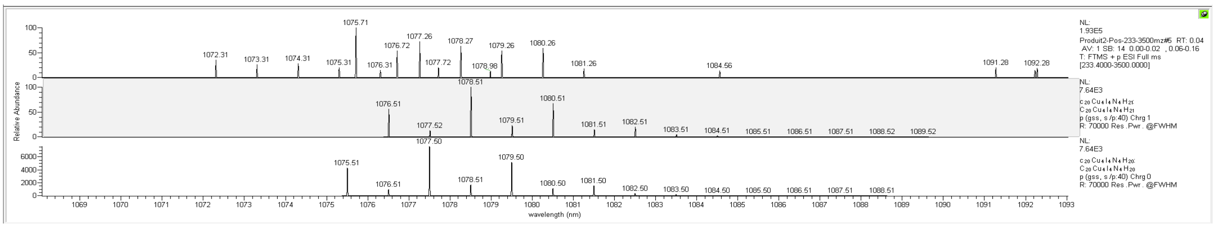

Figure S11. HR-ESI spectrum of the isolated $(CuIPy)_4$ (top), simulated MS spectrum of $[Cu_4I_4(C_6H_5N)_4]+H^+$ (middle), simulated MS spectrum of $[Cu_4I_4(C_6H_5N)_4$ (bottom).
